# Isolated systolic hypertension in young males: a scoping review

**DOI:** 10.1186/s40885-021-00169-z

**Published:** 2021-06-15

**Authors:** Holly Scott, Matthew J. Barton, Amy N. B. Johnston

**Affiliations:** 1 School of Nursing and Midwifery, Griffith University, Nathan, QLD Australia; 2 Australian Army, 2nd General Health Battalion, Townsville, QLD Australia; 3 Menzies Health Institute Queensland, Griffith University, Gold Coast, QLD Australia; 4 School of Nursing, Midwifery and Social Work, The University of Queensland, Woolloongabba, QLD Australia; 5 Department of Emergency Medicine, Princess Alexandra Hospital, Brisbane, QLD Australia

**Keywords:** Blood pressure, Male, Cardiovascular diseases, Hypertension

## Abstract

Isolated systolic hypertension typically occurs in young males; however, its clinical significance is unknown. Given the prevalence of the hypertension and its contribution to global morbidity and mortality, a synthesis of the most recent available evidence around isolated systolic hypertension is warranted. This review aims firstly to review the haemodynamic and physical characteristics indicative of cardiovascular risk in young males (aged 18 to 30 years) with isolated systolic hypertension, and secondly to synthesize the associated clinical management recommendations reported in the literature. Six databases were systematically searched for all relevant peer-reviewed literature examining isolated systolic hypertension in young males. Search results were screened and examined for validity, those that did not meet the inclusion criteria were removed. A total of 20 articles were appropriate for inclusion. Key factors indicative of cardiovascular risk in isolated systolic hypertension were characterized by several distinctive haemodynamic parameters and physical characteristics. After the literature was synthesized based around these key factors, two distinct cohorts (healthy and unhealthy) were highlighted. The healthy cohort of younger males with isolated systolic hypertension was associated with a decreased cardiovascular risk and therefore no medical interventions were recommended. The second (unhealthy) cohort was, however, associated with an increased cardiovascular risk and may therefore, benefit from antihypertensive therapy.

**Supplementary Information:**

The online version contains supplementary material available at 10.1186/s40885-021-00169-z.

## Background

Data published by the World Health Organization suggests that more people die from cardiovascular disease (CVD) than from any other single cause [1]. There is a direct risk of both stroke and ischemic heart disease due to hypertension alone [2]. Globally, CVD is estimated to affect more than 50% of those over the age of 60 [3]. Isolated systolic hypertension (ISH) is a subtype of hypertension. The International Society of Hypertension, defines ISH as having a brachial systolic blood pressure (SBP) greater than 140 mmHg and a brachial diastolic blood pressure (DBP) of less than 90 mmHg [4]. ISH is the most common form of hypertension among both the young and the elderly [5, 6].

In the elderly, ISH is thought to arise as a result of aortic stiffening, this occurs from the normal aging process, in combination with a number of modifiable risk factors (such as smoking and obesity) [5–7]. Increased pulse wave velocity (PWV) in the elderly is an indication of arterial stiffness, a strong determinant of CVD development [8]. Arterial stiffening, observed in the elderly, contributes to increased pulse pressure (PP) which causes further stiffening resulting in end-organ damage [3]. In the elderly, ISH has been clearly linked to increased risk of CVD, and as such, pharmaceutical interventions, such as antihypertensive therapy, offer significant benefits [3]. However, most studies have now concluded that ISH in the young (ISHY; ~ 18–30 years old) is likely the result of a different mechanism to that seen in the elderly.

Some research suggests the presence of ISHY is due to brachial blood pressure augmentation, elastic arteries, or as a result of increase PWV secondary to vascular stiffening, similar to that seen in elderly populations [3, 5]. The European Society of Hypertension published guidelines in 2016 that briefly mentioned the existence of ISHY, however, did little to explain its cause, cardiovascular (CV) risks and management strategies [9]. Diagnosing a young person and treating them for something that has no known associated deficits may have significant lifestyle and career implications [5]. On the other hand, if treatment is indicated, and may minimize long-term CV risk, it should not be withheld. An accurate understanding of the mechanism underpinning ISHY is necessary to define risks associated with it and to guide the clinical management of these young individuals [3]. Preliminary scoping of the literature exploring ISHY suggested that only a small portion was represented by females. For this reason, the principal concept explored in this review was centred around data from ISHY in males, irrespective of the study setting or cultural context.

The priority questions addressed in this review are two-fold; firstly, what is the clinical significance of ISHY in males, and secondly, what clinical recommendations exist in the literature regarding the management of ISHY. These questions informed the aims of this review, namely: (1) identify the key indicators of CV risk associated with ISH in males between 18 and 30 years of age, and (2) determine how ISH can be managed within this cohort. Given the prevalence of hypertension and its contribution to global morbidity and mortality, ISHY certainly warrants attention.

### Design

This scoping review adopted the JBI evidence-implementation approach, as set out by Peters et al. [10].

Preliminary searches for CV risk factors (see Appendix for details [11–15]) and interventions associated with ISH identified a wide range of research designs and outcome measures, ruling out a systematic review with meta-analysis. It was also identified that most established research on ISH was conducted on the elderly. Within the younger population, only a small portion was represented by females and results were, therefore, clinically negligible. For this reason, the review focused on young males as its target population, irrespective of the cultural context or setting (acute, community) in which the data were collected. Generally, scoping reviews are indicated for identifying and analysing knowledge gaps, clarifying concepts and definitions, and mapping evidence to inform practice [16, 17]. Therefore, a scoping review was the methodology selected to afford a broader lens to explore CV risk factors associated with ISH in young males, and respective management recommendations.

### Search methods

A comprehensive literature search of six databases was conducted, including Johanna Briggs, Embase, CINAHL, MEDLINE, Cochrane Library, and PubMed. All the searches were executed in March 2020, several rounds of preliminary searches were conducted as per the systematic scoping methodology to identify the best MeSH terms (descriptors or subject headings), keywords, and to test search strategies [10]. The search terms used included the following MeSH, keywords and Boolean terms: “systolic hypertension” OR “isolated systolic hypertension” AND “young”. Years of coverage were 2000 to 2020. Study participant limiters such as gender and age were applied, and the search strategy was customized to each database.

### Inclusion and exclusion

**Fig. 1:**
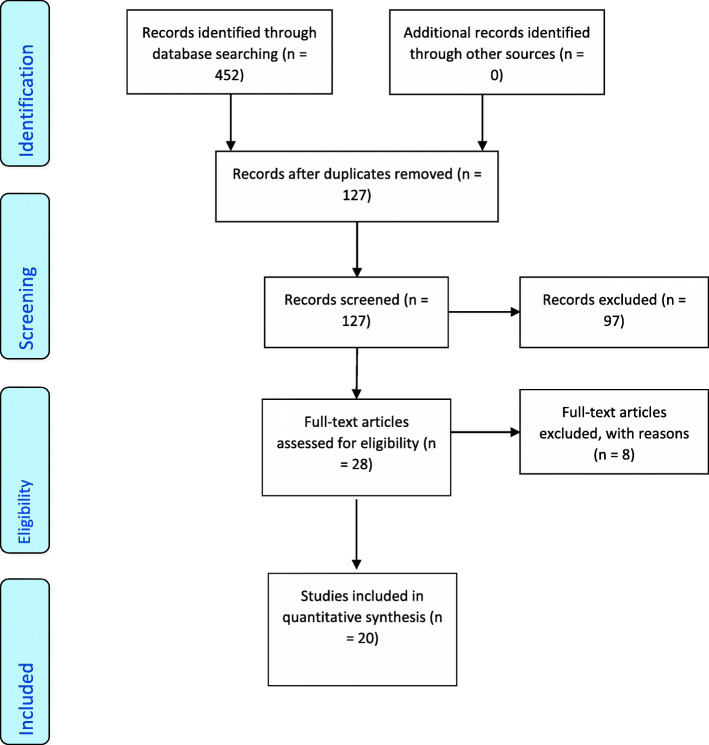
Schematic PRISMA-type illustration of the systematic study selection process undertaken to inform this review

Only literature that met the inclusion criteria were included in the review namely: (1) full-text articles published in the English language within the last 20 years, (2) studies conducted in the young males 19 to 24 years or 19 to 44 years depending on the database limiters, (3) studies that evaluate SBP, and (4) studies published in peer-reviewed journals. Article results that did not differentiate gender and age groups were excluded, as it was not possible to stratify relevant data. Studies assessing cohorts with pre-existing medical conditions we also excluded as ISH could not be examined independently (Fig. 1).

### Data collection and analysis

A total of 452 articles were reviewed by author one and reduced to 127, after the removal of duplicates, were identified that met the initial search criteria (Fig. 1). These were independently reviewed by a second author. Consensus was achieved on all papers. Articles that were not in English, focused on a cohort with pre-existing medical conditions or were not published within the last 20 years were excluded (*n* = 97) (Fig. 1). The remaining 20 full-text articles were used to inform the review. A PRISMA flow diagram (Fig. 1) illustrates the scoping review process [18].

A data extraction table (Table 1) was developed to chart the results. It enabled the extraction and synthesis of the key findings from 14 articles, including: authors, year of publication, study design and population, study aims and methodology, and recommendations/results. Development of these conceptual categories was iterative and undertaken to ensure alignment with the review questions [10]. The authors independently evaluated each of the studies for several characteristics, including study design, aim, sample size, population, methodology and key findings, as per the scoping review framework [10]. Six articles (not included in the extraction table) were used to contextualize, inform and confirm the clinical recommendations and therefore, did not contribute directly to the data extracted from the studies. Evidence from the studies that met the selection criteria was tabulated and then synthesized in the results section to enable the development of an overarching picture of the clinical significance of ISHY in males, and to propose clinical recommendations in regard to the management of ISHY on the basis of synthesis of best available evidence.

**Table 1:** Included papers

Study	Study design	Sample size	Aim	Population	Methodology	Finding
Eeftinck Schattenkerk et al. (2018) [19]	Prospective cohort Study	3744 Participants	Assessed prevalence of ISHY and compared differences in cSBP and arterial stiffness between ISHY and other hypertensive phenotypes.	Large multi-ethnic HELIUS study was used. The cohort age was under 40 years, with the average 29 ± 6 years. 83% were male.	The study assessed the prevalence and haemodynamic parameters (cBP and atrial stiffness) of ISH. These findings were compared with normotensive, SDH, and IDH. Those with diabetes with identified in the HELIUS study.	ISH represented 2.7% of the population (greatest prevalence in African ethnicity), ISH individuals had lower cSBP and PWV compared to IDH or SDH. ISH individuals were taller had lower AIx and larger SV in comparison to other subgroups. Having increased SBP amplitude was associated with being male, Dutch, younger, taller, a lower AIx and an increased SV. Overall, the study indicated that the haemodynamic profile of ISH was similar to that of ‘high-normal’ BP.
Palatini et al. (2018) [20]	Long-term prospective cohort study	1206 Participants	Investigate the risk of hypertension requiring pharmacological treatment in ISH.	209 Individuals between 18 and 45 years. 89.9% were male.	Data was collected from the Hypertension and Ambulatory Recording Ventia Study. Using a 24 h BP monitor, 209 individuals were found to have ISH. Cox survival analyses were used to evaluate the role of ISH in predicting hypertension development.	ISH individuals were more frequently young males active in sport, had a low heart rate and low cholesterol. In a 6.9-year follow-up, 61.1% had developed SDH. ISH had non-significant increase in hypertension compared to the normotensive group; SDH and IDH groups had a significant increase. The study suggested mean BP identified with 24-h ABPM, is required for identification and predictive risk. ISHY identified with 24 ABPM does not imply an increased CVD risk.
Gaddum et al. (2017) [21]	Experimental study	Bovine and silicon models	Characterize individual parameters contributing to arterial pressure and its amplification.	Bovine V silicon model.	Silicon arterial tree was compared to a bovine aorta to study variable SV profiles. Inotropy was altered in the ventricles and arterial parameters were altered to replicate wall thickness, taper, diameter and bifurcation to compare bovine to silicone.	Amplification increased with bifurcation, and CO, decreased with wall thickness and vessel taper. PP increased with wall thickness (stiffness) and taper angle and decreasing diameter. According to the study conclusions, PP amplification is primarily due to ventricles and not arterial stiffness.
Radchenko et al. (2016) [13]	Cohort study	44 Participants	To identify simple clinical predictors for increased cBP in ISHY.	All participants were males, average age of 32.2 ± 1.3 years. Individuals significant comorbidities were excluded.	Measurements including weight, height, office BP, HR, ambulatory BP, PWV, cBP, biochemical blood test, ECG, echo, carotid u/s were included. These measurements were used to identify links to cBP. Organ damage and pathology were also assessed.	Independent predictors of increased cBP included height < 178 cm, weight > 91 kg, DBP > 80 mmHg. The presence of two or more increased the risk of elevated cBP more than ×10. PWV and SVR were significantly higher in ISH individuals with elevated cBP, were as CO and SV were higher with normal cBP. Increased cBP occurred most frequently in those who were older, shorter and had a higher BMI. The study recommended lifestyle modification for ISHY, and not BP medication.
Johnson et al. (2015) [22]	Multi-disciplinary observational study	3003 Participants	Compare the rates of receiving a hypertension diagnosis and antihypertensives among young adults with ISH, IDH, and SDH.	The included participants (61% males) ranged from 18 to 39, each with hypertension. 45% were identified to have ISH.	Participants with a pre-existing diagnosis of hypertension were excluded. Participants were all require to be currently being managed: receiving regular primary care contact (2 office encounters in 3 years, 1 being in the last 2 years).	The study found that 56% with ISH received a diagnosis compared to 63% with SDH. 32% with ISH were given antihypertensives compared with 52% in the SDH cohort. ISH was found to have a 50% poorer diagnosis and 31% treatment rates.
Musinguzi, Van Geertruyden, & Bastianens (2015) [23]	Comparative cross-sectional study	4432 Participants	Identify the prevalence of uncontrolled hypertension.	Participants in two Ugandan districts. Cohorts were divided on age groupings: 15–34, 35–49, 50+. 36.3% were male.	BP measurements were taken three times, at 1 min apart, after the participant had been seated for 5 min. The assessment was conducted in one visit only. All participants that had already achieved hypertension control were excluded.	The study identified the prevalence of uncontrolled hypertension to be a total of 20.2%. ISH represented 7.2% of those who had uncontrolled hypertension. The results were not found to differ between the genders.
Yano et al. (2015) [24]	Retrospective study	39,441 Participants	Assess CVD risk of ISH in young and middle-aged adults.	The study included 15, 868 males and 11,213 females (58.5% male) between the ages of 18–49 from Chicago, USA between 1967 and 1973	Chicago Heart Association Detection Project in Industry study. Participants were classified into the following: (1) SBP < 130 mmHg & DBP < 85 mmHg; (2) SBP 130–139 mmHg and DBP 85–89 mmHg; (3) ISH; (4) IDH; (5) SDH.	Over long-term follow-up, younger and middle-aged adults with ISH had higher relative risk for CVD mortality than those with optimal-normal BP. The study did not recommend antihypertensive medication to reduce risk.
Saladini et al. (2011) [14]	Long-term prospective cohort study	388 Participants	Investigated prognosis of ISH in young-to-middle-aged individuals differs according to cBP.	Participants included 18–45 years from the Hypertension and Ambulatory Recording Ventia Study.	354 Participants with stage 1 hypertension and 34 with normotension to identify which individuals would progress to require antihypertensives. Baseline BP was the mean of six, over two visits that were two weeks apart. ISH-high (high cSBP) and ISH-low (low cSBP) were separated and assessed.	ISH predominately affected younger males. ISH-low was represented by 93.9% males, and ISH-high was composed of 88.2% males. ISH-high was identified to have decreased larger arteries and higher peripheral resistance. ISH-low had similar variables to normotensive. They were identified to have a low-risk of HTN requiring treatment. ISH-low were younger, had a lower BMI, smoked less, had a lower total cholesterol and lower triglycerides than all other groups including normotensive individuals.
Sundstrom et al. (2011) [25]	Nationwide cohort study	1,207,141 Participants	Investigate the nature and magnitude of relations of systolic and diastolic blood pressures in late adolescence to mortality.	All participants were males, Swedish males conscripted to military. The average age was 18.4, with a follow-up age of 24.	Data from Swedish Military Conscription Registry. Individuals were born between 1949 and 1979. 2–3% were exempt due to disability or chronic disease, 17% lost data. The remaining 1,207,141 males had SBP 80–184 and DBP 30–120. The Cox proportional hazards models were used to investigate relations of BP to risk of death.	The study identified 28, 934 males (2.4%) had died. DBP below 90 mmHg was unrelated to mortality, and above which significantly increased risk. The lowest risk identified was found to be associated with a SBP around 130 mmHg. The optimal SBP is unclear however, may correlate with age. The association between SBP and mortality was unclear.
Grebla et al. (2010) [11]	Prospective cohort study	5685 Participants	The study examined the prevalence and determinants of ISH in 18 to 39-year-olds.	Data was collected from three consecutive NHANES reports from 1999 to 2004 (49.6% male).	Participants had prevalence and risk estimated by age and gender, those who were already prescribed antihypertensive medications were removed.	The total prevalence of ISH was 1.57% ± 0.23%, with the greatest prevalence in 18–29 years. ISH was associated with being male, smoker, obese and of a low socioeconomic status. ISH individuals were younger, have a higher PP than all groups and taller than the normotensive group. The study also identified that the prevalence of IDH and SDH increased with age where as ISH decreased.
Hulsen et al. (2006) [26]	Prospective cohort study	750 Participants	Investigated prevalence and determinants of SSH in young adults and their 20-year risk of CHD.	352 Males and 398 Females (46.9% male) aged 26–31. 57 Males has ISH.	Data from the Atherosclerosis Risk in Young Adults study. Measurements included brachial BP and aortic pressures. Central haemodynamic measured with SphygmoCor.	SSH males had a higher BMI, had a significantly higher brachial and cBP, PP and MAP. AIx was significantly lower compared to normotensive individuals. SSH males smoked less, and were taller than all other groups, other characteristics were not significantly different. In conclusion, SSH individuals did not have a statistically significant increased risk of CVD.
McEniery et al. (2005) [27]	Long-term prospective cohort study	1008 Participants	Test the hypothesis that ISHY and essential hypertension (SDH) have different haemodynamic mechanisms.	Participants from the ENIGMA study (49.2% male) who were aged 17–27 (91.4% male).	Participants were randomly selected from 2 UK universities. They had their haemodynamic measurements recorded, those with pre-existing conditions were excluded. Brachial BP was taken 5 min after rest. The SphygomoCor was used to obtain central haemodynamic readings. Subjects were required to complete a lifestyle and medical history questionnaire. The study examined peripheral and cBP, aortic PWV, CO, SV, PVR. Comparisons were then made between the hypertensive subtypes and normotensive group.	The study identified individuals with ISHY to have significantly higher brachial BP, cBP, MAP, aortic PWV, CO, and SV compared to other hypertensive subtype groups. ISH were taller, weighed more, smoked significantly less, exercised more than all groups. There was no difference in PVR, HR, or PP amplification in comparison to normotensive individuals. Compared to SDH, MAP, HR, and PVR were all significantly lower; whereas PP amp, aortic PWV, CO, SV were significantly higher. The study concluded that ISH and SDH have different haemodynamic mechanisms. ISH appears to come from increased SV and/or aortic stiffness where as SDH appears to result from increased PVR.
Mahmud and Feely (2003) [12]	Cohort study	174 Participants	Examine the role of high PP in SSH.	174 Healthy medical students (50% male); average age was 23 ± 0.5 years.	Measurements including brachial BP, aortic BP, arterial wave reflection, PP amplification, height and weight were recorded. BP was assessed after 15 min in supine position. Participants completed questionnaire about smoking, physical activity, alcohol.	ISHY with normal cBP were commonly associated with being tall, active, non-smoking males. These individuals also had normal aortic pressure waveform, slower HR, reduced arterial wave reflection and increased PP amplification. Whereas SDH had reduced amplification and enhanced arterial wave reflection. Findings are thought to be the result of exaggerated first systolic peak in brachial artery waveform. PP amplification is an indication of elasticity and reduces with age. AIx is an indication of atrial stiffness. All ISH participants were physically active and regularly participated in sport.

*ISHY* isolated systolic hypertension in the young, *cSBP* central systolic blood pressure, *HELIUS* Healthy Life in an Urban Setting, *cBP* central blood pressure, *ISH* isolated systolic hypertension, *SDH* systolic-diastolic hypertension, *IDH* isolated diastolic hypertension, *cSBP* central systolic blood pressure, *PWV* pulse wave velocity, *Aix* augmentation index, *SV* stroke volume, *SBP* systolic blood pressure, *BP* blood pressure, *ABPM* ambulatory blood pressure monitoring, *CVD* cardiovascular disease, *CO* cardiac output, *CO* cardiac output, *PP* pulse pressure, *HR* heart rate, *ECG* electro cardio gram, u/s ultrasound, *DBP* diastolic blood pressure, *SVR* systemic vascular resistance, *BMI* body mass index, *HTN* hypertension, *NHANES* National Health and Nutrition Examination Survey, *SSH* spurious systolic hypertension, *MAP* mean arterial pressure, *CHD* coronary heart disease, *PVR* peripheral vascular resistance

A total of 20 full-text articles were used to inform the review. These included large, multi-ethnical prospective cohort studies, cross-sectional studies, an experimental study, an observational study, and reviews. The studies involved a total of 1,266,982 participants aged 18 to 49 years, with data collected from 1969 to 2016 (Table 1). The physical characteristics and haemodynamic parameters of these individuals were assessed to understand the potential CV risk of ISHY in males aged 18 to 30, and how ISHY can be most appropriately managed clinically. Populations studied included Vietnamese, American, Swedish, Ugandan, and European backgrounds. Participants were included from large clinical database studies such as the Hypertension and Ambulatory Recording Venetia Study (HARVEST), the Atherosclerosis Risk in Young Adults study, the ENIGMA study, National Health and Nutrition Examination Survey (NHANES), and the Healthy Life in an Urban Setting (HELIUS) study.

To develop an understanding of CV risk, most studies drew a comparison between normal blood pressure and other hypertension subtypes, including ISH, systolic-diastolic hypertension (SDH) and diastolic hypertension. Radchenko et al. [13] noted that the occurrence of hypertension subgroups increased with age, except for ISH which was the most prevalent in males aged 18 to 29 [11, 22]. In terms of management, only 32% of ISH individuals were found to receive antihypertensives in comparison to 52% of SDH individuals and were less likely to receive a diagnosis [22]. From the included studies, key data fields emerged to determine CV risk. This included haemodynamic findings (PP amplitude, PWV, augmentation index [AIx], cardiac output [CO], and central blood pressure [cBP]), modifiable and nonmodifiable risk factors (weight, height, smoker status, physical activity level); see Appendix for more details on CV risk factors.

### ISHY and CV risk

Haemodynamic measurements were consistently used to determine potential CV risk associated with ISHY. Some common ISHY haemodynamic findings included a normal to low heart rate, increased CO, an increased PP amplitude as well as a decreased AIx [12, 19]. In some cases, there was no significant increase in PP amplitude. ISHY participants were also found to have an increased aortic PWV and decreased brachial PWV [21]. This is indicative of increased arterial compliance and a lack of peripheral vascular resistance (PVR) [21]. Consistent with this finding, McEniery et al. [27], found that in 17- to 27-year-old males, PVR in ISH was comparable to normotensive individuals.

Central PP and cBP appear to be a more accurate and useful indicator than brachial BP, as they provide a better indication of the BP exerted on the major organs [28]. In most cases, ISHY individuals were found to have a cBP classified as high-normal, however, in some cases, it was elevated further, and in other cases, it was found to be low [12, 19, 24, 26]. Radchenko et al. [13], noted that independent risk factors for increased cBP in ISHY, a measurement strongly associated with CV risk, included height less than 178 cm, weight greater than 91 kg and DBP more than 80 mmHg. Having two or more of these factors increased the chance of elevated cBP at least 10-fold. Sundstrom et al. [25], also noted the significance of an increased DBP; their study included a total of 1,207,141 males conscripted to the Swedish military forces. DBP less than 90 mmHg was found to be unrelated to mortality, however above 90 mmHg there was a significant increase [25]. Elevated cBP corresponded to elevated systemic vascular resistance. Saladini et al. [14], identified that ISHY with a low or normal cBP was associated with reduced CV risk. ISHY with low or normal cBP were younger, had a lower body mass index (BMI), lower total cholesterol, low triglycerides and smoked less than all other hypertensive subgroups as well as normotensive individuals.

Three studies suggested ISH is spurious, while four studies concluded that ISH presents a similar risk to that of high-normal BP and thus, conclusive findings remain to be established. Yano et al. [24] concluded ISH individuals that were young or middle-age had an increased CV risk, and estimated the risk to be similar to that of high-normal BP, but did not recommend pharmacological treatment. Eeftinck Schattenkerk et al. [19], also concluded that ISH posed a similar CV risk as having a high-normal BP and identified common features in the population tested that included; being male, taller, Dutch, younger, having a larger stroke volume (SV) and reduced AIx. Grebla et al. [11], agreed that the ISHY cohort was predominately male and younger than those of other hypertension subgroups. They additionally found that ISH individuals in the 18- to 39-year-old cohort had an increased BMI, total cholesterol, SBP, DBP, PP and were more likely to be of a lower socioeconomic status compared to the normotensive cohort. PP was also noted to be substantially higher than all other groups.

Mahmud and Feely [12], studied a group of 174 healthy medical students, 11 of which had ISH. They concluded ISHY was associated with normal cBP, tall, active, non-smoking males, which is consistent with Radchenko et al. [13], who additionally reported ISHY individuals to have a slower resting heart rate. The study suggested that the results may be due to an exaggerated first systolic peak in the brachial waveform. The increased pulse transit time seen in the ISH cohort causes a delayed return from peripheral vessels resulting in pulse wave reflections having the greatest positive impact on the peripheral arteries and the least on the central vessels [12]. McEniery et al. [27] suggested that the elevated systolic value in young males is due to decreased PVR, increased SV and aortic stiffness, in contrast with SDH, that is seen subsequent to increased PVR. This study of 1008 participants ages 17 to 27 years, concluded that ISHY is associated with increased physical activity, less smoking, increased height and weight compared to other cohorts. Palatini et al. [20], also found ISHY predominately occur in healthy, fit, young males with a low resting heart rate. The study additionally reported no increased risk of ISHY individuals developing SDH hypertension in the 6.9-year follow-up assessment. As a result, they concluded that ISHY does not imply increased CV risk [20].

### Cohorts and CV risk

ISHY was associated with positive haemodynamic, modifiable and nonmodifiable factors [12, 20, 27] there are some consistent findings such as ISHY predominately occurs in males with a comparatively higher BMI [11, 14, 19]. Among the inconsistent findings, there have been two notable cohort themes drawn. The first (unhealthy cohort) is that ISHY is characterized by individuals being current smokers, of lower socioeconomic status, decreased physical activity and increased BMI [11]. The second cohort theme (healthy cohort), in complete contrast, is that ISHY is characterized by individuals being taller, more physically active, the youngest in the age bracket, lower blood cholesterol levels, are non-smokers; thus depicted as exceptionally healthy [5, 12, 20, 26]. Correspondingly, the haemodynamic findings also identified two distinct cohorts among ISHY, those with increased cBP and those with normal or low cBP. Those with elevated cBP have been linked to CV risk through physical, social and additional haemodynamic factors; those with normal or low cBP have been associated with healthy lifestyle choices, physical activity and low-risk CV haemodynamic parameters [14, 19].

Despite several studies exploring many elements of ISH in younger male populations, the clinical significance of ISH remains unclear. Some studies have concluded that it is spurious and does not require intervention, while others have concluded it is related to increased CV risk. The findings appear to be influenced by the age of participants studied and the cohort size. Cohorts that included males up to 49 years of age were more likely to identify greater CV risk associated with ISH than studies of a younger cohort. Larger cohorts were also more likely to find negative rather than positive CV association with ISH. Over the last decade, the incidence of ISH has increased [29]. This highlights the need for clear evidence around management and consistent, evidence-based, recommendations for the management of ISHY.

### Trajectory and CV risk

Throughout the scoping review, the prevalence of ISH was often described as being represented by a J-curve [5, 7]. This supports the notion that the pathophysiology of ISH differs between the young and the elderly. While ISHY may develop into SDH, evidence suggests most people do not maintain ISH throughout life [5, 7]. Ultimately, individuals need to be traced to determine whether ISHY progresses into SDH and whether they are at an increased risk of specific CV events.

### Haemodynamic findings and CV risk

To review the potential CV risk associated with ISHY, the haemodynamic findings were analysed and summarised in Table 1. Briefly, increased PWV and both normal and increased PP amplification, are noted in ISHY [13, 27], however, ISHY is rarely associated with an increased heart rate [5]. Increase velocity may occur secondary to increased CO which has been noted in ISHY, and increased distance which is also seen in the taller stature of ISHY individuals [12, 19, 21, 27, 30]. PP demonstrated negative CV effects in the elderly, but not in younger populations [31]. Indeed, increased PP amplitude, a common finding in ISHY, may have positive as opposed to negative CV implications, as seen in the elderly [5].

Increased CO, a feature of ISHY, has also been noted in overweight and obese individuals (a modifiable CV risk factor), regardless of their SBP [3]. SBP in these individuals does, however, correlate with increased PVR [3], unlike ISHY in healthy body weight ranges [3, 27]. This suggests that increased CO and SBP in ISHY who are overweight may have a different causative mechanism [3].

ISH in combination with increased heart rate results in increased arterial stiffness and thus an overall increase in CV risk [5], this may be due to a neurogenic abnormality involving sympathetic nervous system dysregulation [32]; referred to as a ‘hyperkinetic circulation’ [5], and associated with an elevation in norepinephrine levels [33]. It is unknown whether this hyperkinetic state precedes sustained SDH. If this adrenergic activation was responsible for ISHY it would likely result in adverse effects [5]. An increase of 10 beats/min would cause an increase in PWV of 0.17 m/sec resulting in an increase in vascular aging by 2 years at 40 years old, however, at the age of 20, this would equate to 5 years of vascular aging [5]. In most cases, ISHY individuals were found to have a heart rate similar or lower than the comparative normotensive cohorts [20].

### Completeness and applicability of evidence

ISHY primarily occurs in males with an increased BMI. However, there were two groups of common findings associated with participant profiling. The first cohort (profile) was characterized by being a smoker, having a lower socioeconomic status and being less physically active. The second cohort was characterized by being taller, more physically active, the youngest in the age bracket, having a low cholesterol, being non-smokers and depicted as exceptionally healthy [12, 20]. This first, less healthy cohort, has haemodynamic findings associated with increased CV risk, such as increased cBP and increased PVR. The second healthy cohort has haemodynamic findings associated with reduced CV risk, including reduced PVR and low or normal cBP [14, 19]. Ascertaining the overall risk of ISHY becomes more difficult as these two opposing cohorts are, perhaps unknowingly, grouped and analysed together.

To obtain a complete understanding of ‘true’ ISHY, these two cohorts, of healthy and unhealthy males aged 18 to 30 years, need to be studied independently. Their haemodynamic characteristics including cBP, brachial BP, aortic PWV, brachial PWV, PP amplitude, AIx, SV, heart rate; as well as the physical characteristics and overall health of the individual must be examined. Variables including pre-existing medical conditions and familial history of CVD should be normalized to appreciate the effect of ISHY. Additionally, participants should also be followed long-term.

### Management of ISHY

**Fig. 2:**
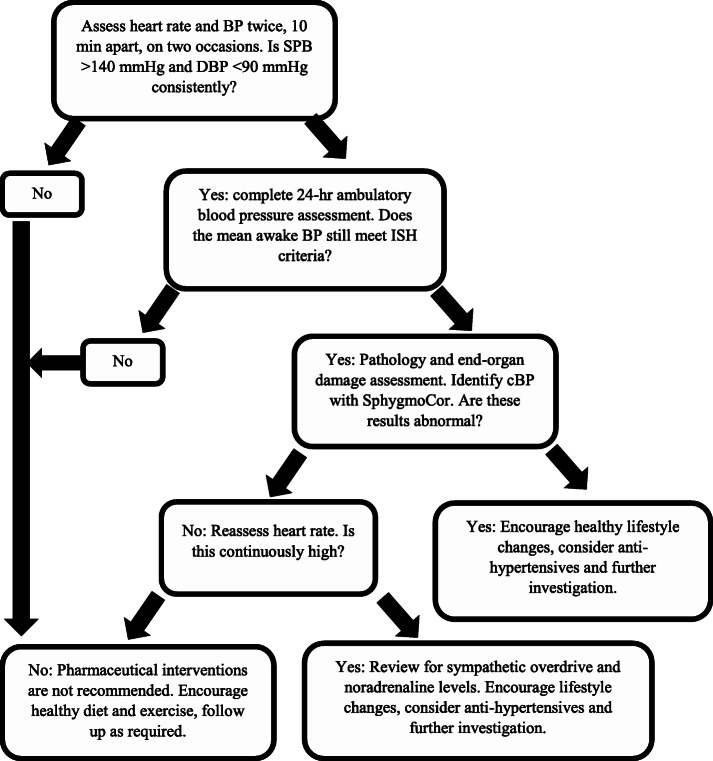
Proposed clinical management of suspected isolated systolic hypertension (ISH) in males 18 to 30 years of age. BP, blood pressure; SBP, systolic blood pressure; DBP, diastolic blood pressure; cBP, central blood pressure

Analysis of the available literature supports several clinical recommendations that may assist in managing ISHY. Individuals aged 18 to 30 with suspected ISH should first have their BP taken in a seated position for consistency, ideally in a non-stressful environment, after no recent physical exertion, to avoid false positives [34]. The heart rate and BP should be assessed twice in the first visit, 10 min apart, to improve accuracy [34]. If one of these blood pressure readings is high, the individual should return one to 2 weeks later to repeat the first visit. If the SBP remains above 140 mmHg, a 24-h ambulatory BP monitor should be fitted. This is necessary to eliminate white coat hypertension (WCH). If the mean SBP during the awake hours remains high, the individual should be assessed for end-organ damage and routine pathology including cholesterol [5]. cBP, evaluated with the SphygmoCor, is a more accurate indicator of CV risk than brachial BP [19]. If pathology, cBP and heart rate are normal, antihypertensive medications are not indicated [5]. Pharmaceutical management of BP may be expensive and can induce secondary effects, and restrict engagement in some activities, which in turn, may impact the quality of life and reduce physical fitness [3]. If cBP is low or normal and heart rate is elevated, assessing for an increased sympathetic drive should be considered [5]. If pathology is abnormal, there is an indication of end-organ dysfunction, or if cBP is elevated, there is a potential that antihypertensive medications could reduce CV risk in this group. A flowchart (Fig. 2) of proposed actions has been devised to summarise the management of ISHY, based on the literature used in the review.

### Consideration of pharmaceutical treatment

Based on this review of literature, treatment is not indicated in ISHY who are healthy and have otherwise normal haemodynamic findings. In high-risk individuals with ISH, antihypertensives may be considered. The therapeutic benefit of B-blockers is unlikely to outweigh the adverse effects in the cohort of young males [3]. McEniery et al. [3], suggested reducing CO as an option to reduce SBP and therefore the potential progression of hypertension, however, the reduced PVR observed in ISHY would challenge this recommendation. Increased CO in ISHY, of healthy body weight, has not been proven to have a negative CVD effect.

### Consideration of non-pharmaceutical treatment

Healthy lifestyle choices, such as weight loss, dietary interventions (reduced sodium intake), and increased physical activity are all recommended approaches in the management of ISHY [35]. A meta-analysis by Neter et al. [36] demonstrated that a 5-kg weight loss and/or physical activity, in adults under 45 years of age, led to a 5 mmHg SBP reduction. While the Dietary Approaches to Stop Hypertension (DASH) diet resulted in a significantly lower SBP at every sodium level (high, intermediate, and low levels) and in a significantly lower DBP at the high and intermediate sodium levels [37].

### Limitations

The scoping review was limited by the number of studies included, and that one author initially searched and compiled the literature. This was an unfunded study and so despite that this is clearly an international clinical issue, only research published in English was included in the review. A significant limitation in many of the reviewed studies was the use of BMI to determine the healthy weight of an individual. BMI is widely known to be a poor indicator of total body adiposity [38]. Muscular individuals may be classified as being overweight, using a BMI scale, due to their muscular weight as opposed to excess adiposity. In order to improve this in future studies, an alternative method should be used, such as the fat mass index or an assessment that incorporates height, hip and waist ratio [37]. A further limitation is that not all studies utilized a 24-h ambulatory blood pressure monitor to differentiate ISH from WCH. This is essential as a large portion of suspected ISHY is WCH. Another significant limitation of these studies was the accurate representation of the young male population; most studies incorporate participants who were middle-aged, and this misrepresents ISHY. Additionally, findings may be impacted by differential impacts of race on blood pressure, however this data is not yet available when looking solely at the young male cohort. The last significant limitation was the lack of adherence to identifying and acknowledging all individuals with pre-existing health conditions or relevant familial medical history. Consequently, the studied cohorts may not have been entirely healthy (or similar) to start with, and the CV risk of ISH could not be independently examined.

## Conclusions

ISHY is associated with decreased AIx, decreased brachial PWV, increased aortic PWV, increased CO and increased or normal PP amplitude. What has been made clear through this review is that there are two distinct cohorts within the ISHY population. The first is characterized by healthy modifiable and nonmodifiable features, including haemodynamic profiling, and the second subgroup has demonstrated physical characteristics and haemodynamic profiling consistent with poor health and may be at an increased CV risk. An ISHY management plan, based on these data has been proposed and could be explicitly validated in future research.

## Supplementary Information

**Additional file 1.**

## Data Availability

Published data over last 20 years.

## Abbreviations

Aix: Augmentation index
BMI: Body mass index
BP: Blood pressure
cBP: Central blood pressure
CO: Cardiac output
CV: Cardiovascular
CVD: Cardiovascular disease
DBP: Diastolic blood pressure
ISH: Isolated systolic hypertension
ISHY: Isolated systolic hypertension in the young
PP: Pulse pressure
PVR: Peripheral vascular resistance
PWV: Pulse wave velocity
SBP: Systolic blood pressure
SDH: Systolic-diastolic hypertension
SV: Stroke volume
WCH: White coat hypertension
