# Supplementary material for: Isolated systolic hypertension in young males: a scoping review
Source: Clin Hypertens. 2021 Jun 15;27:12. doi: 10.1186/s40885-021-00169-z (PMC8204426; doi:10.1186/s40885-021-00169-z)
Supplement: Supplementary file 1 — Additional file 1. [file 40885_2021_169_MOESM1_ESM.docx]

**Appendix**

**Cardiovascular risk factor terminology**

Cardiovascular (CV) risk factors are complex and include several physiological parameters and modifiable risk factors, including weight, level of physical activity, socioeconomic status, smoker status; and nonmodifiable risk factors, including height [11–14]. Haemodynamic parameters can also be used to determine CV risk. These parameters include augmentation index (AIx), pulse wave velocity (PWV), pulse pressure (PP), peripheral blood pressure, central blood pressure (cBP), mean arterial pressure (MAP), stroke volume (SV), and cardiac output (CO) [5]. Arterial stiffening, typically measured using PWV, occurs at differing rates throughout the body and is linked to the development of vascular disease [5]. PP is determined by SV, aortic stiffness and the timing of the pulse wave reflection [5]. PP amplitude is defined as the amount of amplification between the central PP and peripheral PP, this value is influenced by velocity, distance and heart rate [15]. Pulse wave reflection is used to determine the AIx. A combination of CO and peripheral vascular resistance are used to determine MAP [5].
